# Early-life cumulative exposure to excess bodyweight and midlife cognitive function: longitudinal analysis in three British birth cohorts

**DOI:** 10.1016/S2666-7568(24)00005-9

**Published:** 2024-03

**Authors:** Scott T Chiesa, Tom Norris, Victoria Garfield, Marcus Richards, Alun D Hughes

**Affiliations:** a Medical Research Council Unit for Lifelong Health and Ageing at UCL, Institute of Cardiovascular Science, UCL, London, UK; b Division of Surgery and Interventional Science, Institute of Sport, Exercise and Health, UCL, London, UK

## Abstract

**Background:**

Excess bodyweight (BMI >25 kg/m^2^) in midlife (age 40–65 years) has been linked to future cognitive decline and an increased risk of dementia. Whether chronic exposure to excess bodyweight in the early decades of life (<40 years) is associated with compromised cognitive function by midlife, however, remains unclear. This study therefore aimed to test potential bidirectional direct and indirect pathways linking cumulative exposure to excess bodyweight and cognitive function in the early decades of life.

**Methods:**

In this longitudinal analysis, harmonised measures of BMI and cognitive function were available in 19 742 participants aged 47–53 years recruited to the 1946 National Survey of Health and Development (n=2131), the 1958 National Child Development Study (n=9385), and the 1970 British Cohort Study (n=8226). Individual BMI trajectories spanning three decades from age 10–40 years were created for each participant and excess bodyweight duration, BMI change between ages, and cumulative excess bodyweight exposure were calculated. Harmonised measures of verbal and non-verbal ability, mathematical ability, and reading ability were used to create a latent factor for childhood cognitive function, and immediate and delayed recall, animal naming, and letter-search speed tests were used for midlife cognitive function. Multivariable linear regression and structural equation models (SEM) were used to test for potential bidirectional relationships between cognition and excess bodyweight in both individual cohorts and pooled datasets while accounting for other potential early-life confounders.

**Findings:**

Increases in BMI during adolescence and greater cumulative exposure to excess bodyweight across early life were associated with lower midlife cognitive function in all cohorts (eg, pooled difference in cognitive function per 10 years excess bodyweight duration –0·10; 95% CI –0·12 to –0·08; p<0·001). Further adjustment for childhood cognitive function attenuated many of these associations towards the null (eg, pooled difference in cognitive function per 10 years excess bodyweight duration –0·04; 95% CI –0·06 to –0·02; p=0·001), however, with any remaining associations then fully attenuating once further adjusted for other early-life factors (eg, pooled difference in cognitive function per 10 years excess bodyweight duration 0, –0·03 to 0·01; p=0·38). In the reverse direction, low childhood cognition was associated with greater cumulative exposure to excess bodyweight over the next four decades, although much of this relationship was found to probably be explained via other potentially modifiable upstream early-life factors such as childhood disadvantage. SEM in all cohorts suggested the presence of modest direct and indirect pathways connecting earlier cognitive function to later excess bodyweight, but scarce evidence for an effect of early-life excess bodyweight on cognitive function by midlife.

**Interpretation:**

The association between cumulative exposure to excess bodyweight in early life and lower cognitive function in midlife is probably confounded by a persistently lower cognitive function from childhood. Initiatives to improve early-life factors such as childhood disadvantage and education, however, might exert dual but independent benefits on both of these factors before old age.

**Funding:**

Alzheimer's Research UK, Diabetes Research and Wellness Foundation, Diabetes UK, British Heart Foundation, and Medical Research Council.

## Introduction

Dementia is one of the leading causes of morbidity and mortality in the world today, leading to an estimated 1·6 million deaths worldwide in 2019 alone.^1^ Forecasts have predicted that the number of people living with the disease will more than double over the next 25 years, from approximately 60 million today to more than 150 million by 2050.^2^ Although trials of pharmacological treatments such as lecanemab and donanemab have shown some success in the slowing of late-stage cognitive decline and clinical disease,^3^, ^4^ recent years have also seen an increased focus on prevention strategies targeted earlier in the lifespan as a means of reducing disease burden before old age (>65 years).

**Research in context**

**Evidence before this study**

Excess bodyweight in midlife (40–65 years) is associated with an increased risk of dementia in later life (>65 years). Although much research has focused on prospective rates of cognitive decline in individuals who are middle aged with excess bodyweight as they transition to older age, relatively little attention has been paid to the potential impact that decades of chronic exposure to excess bodyweight before this point might have already had on cognitive function. Furthermore, where associations between these factors have been shown, it remains unclear as to which way this causal relationship might operate (ie, higher excess bodyweight causing increased risk of low cognitive function or lower cognitive function increasing risk of gaining excess bodyweight), or whether any associations might instead be explained by other early-life factors such as childhood disadvantage or education. We searched Google Scholar and PubMed from inception to June 1, 2023, for references addressing early-life excess bodyweight and cognitive function in large population cohorts. The search terms we used included “early life” OR “young adulthood” OR “youth” OR “adolescence” OR “child” OR “childhood” AND “overweight” OR “obesity” OR “obese” OR “body mass index” OR “BMI” OR “adiposity” AND “cognition” OR “cognitive” OR “cognitive ability” OR “cognitive function” OR “intelligence” OR “IQ” OR “g factor”.

**Added value of this study**

Using harmonised data collected longitudinally across three separate and long-running national birth cohorts, we aimed to test and then replicate potential bidirectional associations between cumulative exposure to excess bodyweight and cognitive function in early life. We found that previously reported relationships between excess bodyweight and cognitive function before midlife are probably confounded by a persistently lower cognitive function from childhood, whereby children with low cognitive function are more likely to gain excess bodyweight earlier and for longer rather than vice versa. Much of this relationship, however, is likely to be explained by confounding or mediation by other early-life factors such as childhood disadvantage and education.

**Implications of all the available evidence**

Our data suggest that a failure of many dementia studies to account for pre-existing midlife cognitive differences that arise from early-life sociological and developmental factors might result in undue importance being ascribed to proximate associations observed in old age. Although our findings suggest that interventions to reduce excess bodyweight exposure in the early decades of life are unlikely to affect cognitive development and function by midlife, they add to a growing body of evidence suggesting that initiatives to improve early-life sociological factors such as childhood disadvantage and education might exert lifetime benefits on several health outcomes, both physical and cognitive, from the very early years.

It is now estimated that up to 40% of dementias worldwide might be accounted for by 12 common and potentially modifiable risk factors.^5^ One of the most prominently studied of these is midlife excess bodyweight (BMI >25 kg/m^2^),^6^ with observational studies repeatedly demonstrating associations between elevated BMI in middle age (40–65 years) and an increased risk of dementia later in life (>65 years).^7^ The majority of studies investigating potential reasons for this link to date have predominantly focused on changes occurring during the transition from midlife to late life, with many, but not all, studies reporting modestly accelerated rates of cognitive decline over this time frame.^8^, ^9^, ^10^ Much less attention, however, has been paid to the potential effect that a sustained exposure to excess bodyweight in the decades preceding midlife might have on cognitive development and function before this point. Since the 1970s, substantial increases in childhood obesity rates have occurred, and estimates now suggest that up to 80% of children with obesity are likely to still have obesity by midlife.^11^ It can therefore reasonably be expected that the majority of individuals classified as being overweight or obese in midlife have probably already been chronically exposed to excess bodyweight for many years, if not decades, before this point.

Although numerous studies have reported associations between excess bodyweight and cognitive function early in the lifespan, the majority have been limited by a combination of cross-sectional study designs, inability to account for chronic exposure over time, scarcity of data accounting for potentially confounding early-life factors, and the absence of reproducibility across populations. Furthermore, the directionality and causality of this relationship has also been called into question, with some longitudinal studies at this age suggesting that excess bodyweight in midlife might in fact be a consequence of low cognitive function in early life, and others suggesting that any associations seen might be the result of confounding by other childhood factors.^12^, ^13^

By using data from three longitudinal British birth cohorts with up to six repeat measures of BMI across early life, harmonised measures of cognitive function in both early life and midlife, and the availability of harmonised data addressing several potentially confounding physiological and sociological factors across the early lifespan, this study therefore aimed to test potential bidirectional direct and indirect pathways linking cumulative exposure to excess bodyweight and cognitive function in the early decades of life.

## Methods

### Cohort descriptions

For this longitudinal analysis, data from a total of 19 742 participants aged 47–53 years were drawn from three ongoing longitudinal birth cohorts: the 1946 Medical Research Council National Survey of Health and Development (NSHD); the 1958 National Child Development Study (NCDS); and the 1970 British Cohort Study (BCS70). Only participants with cognitive outcomes available in midlife (age 53 years in the NSHD, age 50 years in the NCDS, and age 53 years in the BCS70) were included in the analysis. Further details on cohort participant numbers and recruitment strategies can be found in the appendix (pp 2, 16). All studies have ethical approval for ongoing secondary analyses.

### Childhood cognitive function

Childhood cognitive function was derived as a latent *g* factor underlying four harmonised cognitive tests of verbal and non-verbal reasoning, mathematics ability, and reading ability, all measured at ages 10–11 years. Further details on these tests and the harmonisation process have been previously published (appendix pp 2–3).^14^

### Serial BMI exposures

**Figure 1:**
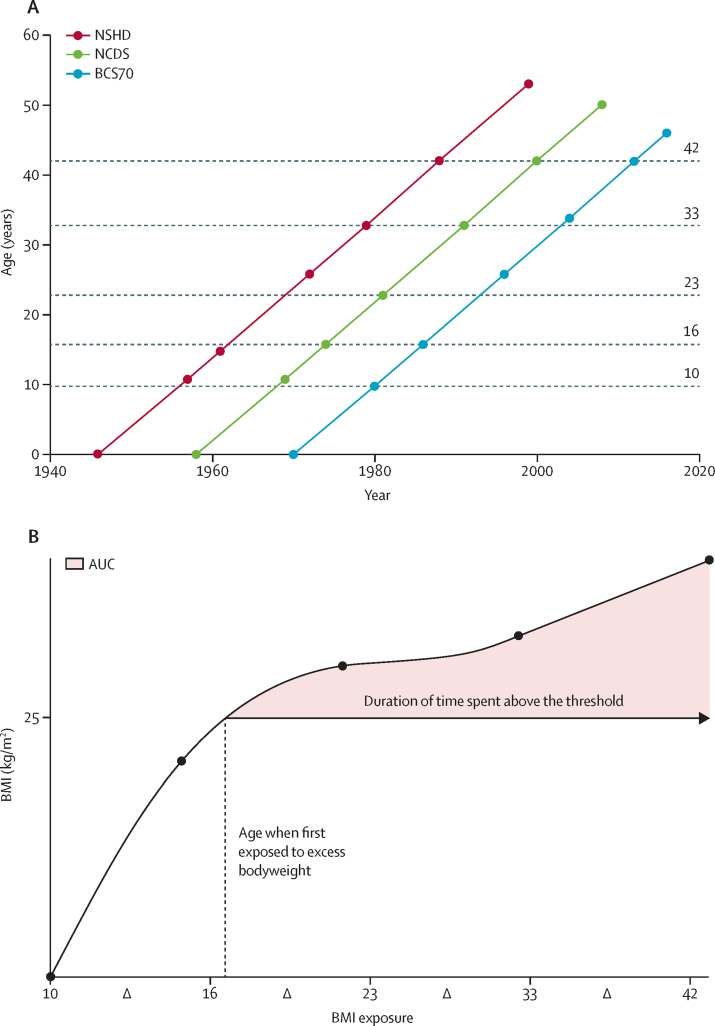
Study timeline and data collection (A) Timeline of study visits in all three cohorts from conception to follow-up, with each dot representing a point at which BMI was either directly recorded or self-reported (top panel). (B) Example of individual child–adult BMI trajectory from which duration of and cumulative exposure to excess bodyweight can be calculated. Delta signifies change in BMI between respective ages. AUC=area under the curve for cumulative exposure to excess bodyweight. BCS70=1970 British Cohort Study. NCDS=1958 National Child Development Study. NSHD=1946 Medical Research Council National Survey of Health and Development.

BMI was derived as (weight in kg/height in m^2^) from either direct measures or self-reporting as follows: at ages 11 years, 15 years, 26 years (self-reported), 33 years, 42 years, and 53 years in the NSHD; 11 years, 16 years, 23 years (self-reported), 33 years, 42 years (self-reported), and 50 years in the NCDS; and 10 years, 16 years (a third self-reported), 26 years (self-reported), 34 years (self-reported), 42 years (self-reported), and 47 years in the BCS70. For the purposes of cross-cohort comparisons, these timepoints are referred to as ages 10 years, 16 years, 23 years, 33 years, 42 years, and 50 years in all cohorts from this point on (figure 1B).

### Midlife cognitive outcomes

Four retrospectively harmonised cognitive tests comprising an animal-naming test, letter-search test, and both immediate and delayed recall tests were done at age 53 years in the NSHD, age 50 years in the NCDS, and age 47 years in the BCS70 (appendix p 4). These were subsequently used to derive an underlying latent *g* factor representing midlife cognitive function, which has previously been shown to test comparable constructs across each of the included cohorts.^14^

### Covariates in early life

Parents’ BMI, child's birthweight, childhood household overcrowding, childhood socioeconomic status (assessed using the father's occupation), and highest education level attained were included as potentially confounding factors in fully adjusted analyses because of the known associations between early-life developmental and family background factors and both weight status and cognitive function (appendix p 4).^15^

### Statistical analysis

Continuous data were summarised as mean (SD) or median (IQR) if skewed. Testing for sex interactions in pooled data yielded little evidence of effect modification and so all analyses were done with sexes combined to maximise power. For our initial analyses, multivariable linear-regression models were used to assess associations between four excess bodyweight exposures (early-life measures of BMI at each clinic visit, BMI change between clinic visits, duration of excess bodyweight, and cumulative exposure to excess bodyweight) and cognitive function in midlife in each of the three cohorts. For BMI at each clinic visit, all measures were standardised to age-specific Z scores before inclusion in models. For BMI change between clinic visits, BMI at the later age was regressed against the previous age and the residuals of this model subsequently used as exposures in future analyses. To create our two metrics of longitudinal excess bodyweight exposure across early life, individual child-to-adulthood trajectories of BMI spanning a range of three decades from ages 10 years to 40 years were created (figure 1B), as previously described in detail in the literature,^16^ and briefly described in the appendix (p 3). All multivariable regression models were assessed using the following three levels of adjustments: model 1 was equal to adjustments for age and sex; model 2 was equal to model 1 plus additional adjustment for childhood cognitive function; model 3 was equal to model 2 plus additional adjustments for parents BMI, child's birthweight, childhood household overcrowding, childhood socioeconomic status, and highest educational attainment achieved. Data from all three cohorts was also pooled and multivariable linear models employing the same models as before but with cohort additionally included as a covariate were used to test the same relationships. Multiple imputations using chained equations (50 imputed datasets, determined by calculation of fraction missing information times 100) were used to account for missing data in statistical models.^17^ Details of the proportion of data missingness are shown in the appendix (p 6). Next, we used childhood cognitive function split into tertiles as an exposure and tested its association with BMI at different ages as an outcome. BMI z-scores were created at all clinic visits (ie, at ages of about 10 years, 16 years, 23 years, 33 years, 42 years, and 50 years) and were then regressed against childhood cognitive tertiles. A simple line graph was then created to graphically approximate trajectories of BMI deviations from expected norms at various timepoints across early life while adjusting for sex. These models were then repeated again with further adjustments for other early-life factors lying upstream of childhood cognition that might have potentially affected the findings, namely parents’ BMI, child's birthweight, childhood household overcrowding, and childhood socioeconomic status. Finally, cross-lagged structural equation models (SEM) were constructed in each of the cohorts to more robustly test potential bidirectional relationships between excess bodyweight and cognitive function across the first half of life. SEM are advantageous in that it allows the simultaneous estimation of both direct and indirect pathways between latent and observed variables. For example, although associations between excess bodyweight in childhood and cognitive function in midlife might arise through early-life developmental differences (direct effect), they might also arise because of the tendency for children with excess bodyweight to grow into adults with excess bodyweight, with adult excess bodyweight then affecting cognitive function during adulthood (indirect effect). Furthermore, the temporal ordering of variables in the model (ie, sequential occurrence of events over time) and availability of repeat measures of potential exposures and outcomes allows the direction of association between all included variables to be inferred. All SEM were adjusted for sex, childhood household overcrowding, and childhood socioeconomic status. Maximum likelihood with missing values was used to account for missing data and all effect sizes were reported as standardised coefficients and SEs. Model fit was assessed using a combination of the root mean square error of approximation (RMSEA; values <0·1 deemed to be a good fit), comparative fit index (CFI; values >0·9 deemed to be a good fit), and Tucker-Lewis Index (TLI; values >0·9 deemed to be a good fit). All analyses were done using StataMP 18 or R version 3.5.3 An a-priori decision was made to interpret findings mainly on the basis of model estimates and their 95% CIs rather than assign significance using an arbitrary p value cutoff of 0·05. These p values are still highlighted throughout, however, for reference.

### Role of the funding source

The funders of the study had no role in study design, data collection, data analysis, data interpretation, or writing of this report.

## Results

Demographics were broadly similar across all three cohorts, with each consisting of roughly equal numbers of male and female individuals and being almost exclusively of White ethnicity (97–100%; table 1). There was some evidence of cohort effects for BMI, with later cohorts, particularly BCS70, showing evidence of an earlier onset and more severe exposure to excess bodyweight across early life, as has previously been reported.^18^ Participant characteristics from the cohorts were similar between those included and excluded from this study with the exception of the highest level of education obtained which was higher in included participants (appendix p 7).

**Table 1:** Participant characteristics

		**NSHD**		**NCDS**		**BCS70**	
		n	Data	n	Data	n	Data
**Characteristics**							
Age at midlife cognitive assessment (years)		2131	53 (0)	9385	50 (1)	8226	47 (1)
Sex							
	Female	2131	1039 (49%)	9385	4771 (51%)	8226	4261 (52%)
	Male		1092 (51%)		4614 (49%)		3965 (48%)
Ethnicity*							
	White	2131	2131 (100%)	9072	8891 (98%)	7129	6907 (97%)
	Non-white		0		181 (2%)		222 (3%)
**Early-life measures**							
Mother's BMI (kg/m^2^)		1855	23·0 (20·7–25·8)	7834	23·0 (21·3–25·6)	6979	22·5 (20·9–24·8)
Father's BMI (kg/m^2^)		1461	23·7 (22·1–25·8)	7617	24·5 (22·6–26·4)	6645	24·0 (22·4–25·9)
Birthweight (kg)		2122	3·4 (0·5)	8606	3·3 (0·5)	6645	3·3 (0·5)
Household overcrowding (people per room)		2011	..	7914	..	6932	..
	≤1	..	1152 (57%)		4774 (60%)	..	5682 (85%)
	>1 to 1·5	..	538 (27%)		2148 (27%)	..	806 (12%)
	>1·5 to 2	..	194 (10%)		764 (10%)	..	143 (2%)
	>2	..	127 (6%)		228 (3%)	..	31 (1%)
Socioeconomic status (father's occupation)		1822	..	6360	..	6507	..
	Unskilled, V	..	143 (8%)	..	290 (5%)	..	253 (4%)
	Partly skilled, IV	..	348 (19%)	..	871 (14%)	..	810 (12%)
	Skilled, III	..	888 (49%)	..	3390 (53%)	..	3485 (53%)
	Managerial and technical, II	..	338 (18%)	..	1417 (22%)	..	1449 (22%)
	Professional, I	..	105 (6%)	..	392 (6%)	..	510 (8%)
Cognitive measures							
	Verbal reasoning	1837	30 (11)	8145	29 (11)	6023	30 (6)
	Non-verbal reasoning	1837	28 (9)	8145	28 (9)	6034	29 (9)
	Mathematical ability	1833	27 (11)	8144	23 (13)	6097	32 (8)
	Reading ability	1832	30 (7)	8144	24 (9)	6635	31 (5)
Highest educational level achieved		1580	..	7974	..	7119	..
	Lower than ordinary secondary	..	695 (44%)	..	1725 (22%)	..	2190 (31%)
	Ordinary secondary	..	315 (20%)	..	3917 (49%)	..	2732 (38%)
	Advanced level	..	425 (27%)	..	1208 (15%)	..	510 (7%)
	Higher education	..	145 (9%)	..	1124 (14%)	..	1687 (24%)
**BMI measures**							
Age 10 years (kg/m^2^)		1788	17·0 (15·8–18·4)	7399	16·9 (15·8–18·5)	6476	16·5 (15·5–17·9)
Age 16 years (kg/m^2^)		1624	19·7 (18·2–21·5)	6705	20·2 (18·8–22·0)	3521	20·7 (19·2–22·8)
Age 23 years (kg/m^2^)		1676	22·0 (20·4–23·8)	7860	22·1 (20·5–24·0)	4666	23·0 (21·1–25·4)
Age 33 years (kg/m^2^)		1917	23·8 (21·8–26·2)	7864	24·3 (22·2–27·1)	6520	25·0 (22·6–28·0)
Age 42 years (kg/m^2^)		1987	24·8 (22·7–27·6)	8561	25·2 (22·8–28·1)	6199	25·9 (23·3–29·3)
Age 50 years (kg/m^2^)		2110	26·7 (24·2–29·9)	7533	26·7 (24·0–30·1)	7177	27·8 (24·6–31·5)
**Cumulative exposure to excess bodyweight measures**							
Ever exposed to excess bodyweight		2121	885 (42%)	9352	4750 (51%)	8136	4908 (60%)
Age when first classed as having excess bodyweight (years)		885	28 (21–34)	4750	26 (21–32)	4908	26 (20–31)
Total duration of excess bodyweight (years)		885	11 (5–18)	4750	14 (7–19)	4908	14 (9–20)
Severity of excess bodyweight (BMI-years)		774	10 (5–17)	4716	18 (4–50)	4895	26 (8–68)
**Midlife measures**							
Cognitive measures							
	Animal naming	2131	24 (7)	9385	22 (6)	8226	24 (6)
	Immediate recall	2131	6 (2)	9385	7 (1)	8226	7 (1)
	Delayed recall	2131	8 (2)	9385	5 (2)	8226	5 (2)
	Letter search speed	2131	279 (76)	9385	334 (89)	8226	346 (85)

Data are presented as n (%), mean (SD), and median (IQR). Severity is expressed as BMI-years and incorporates both duration of time spent exposed to excess bodyweight and the extent to which BMI exceeded the threshold over this timeframe. NSHD=1946 Medical Research Council National Survey of Health and Development. NCDS=1958 National Child Development Study. BCS70=1970 British Cohort Study.

* Ethnicity data were only available as a binary white or non-white variable.

Minimally adjusted models (age and sex only, model 1) showed broadly consistent associations between BMI in early life and overall cognitive function in midlife across all three cohorts (table 2). In brief, larger gains in BMI during the transition through adolescence and young adulthood (ages 10–33 years) and higher BMI from young adulthood onwards (ages 33–50 years) were broadly associated with an approximately 0·1 SD lower overall cognitive function in midlife in each of the individual cohorts (roughly equivalent to an IQ difference of 1–2 points). These modest associations, however, were substantially attenuated (model 2) following adjustment for childhood cognitive function (entirely towards the null in NSHD and NCDS cohorts) and attenuated to the null in all cohorts once adjusting for additional early-life covariates (model 3). A similar pattern was observed when using pooled data (appendix p 8) and when using each of the four individual cognitive tests (ie, animal naming, immediate recall, delayed recall, and letter-search tests) as outcomes (appendix pp 9–10).

**Table 2:** Associations between early-life measures of BMI and midlife cognitive outcomes

	**NSHD (n=2131)**			**NCDS (n=9385)**			**BCS70 (n=8226)**		
	Model 1	Model 2	Model 3	Model 1	Model 2	Model 3	Model 1	Model 2	Model 3
**BMI at distinct ages**									
Age 10 years	0·05 (−0·01 to 0·12)	0·05 (−0·01 to 0·10)	0·05 (0·00 to 0·11)	0·01 (−0·02 to 0·04)	0·00 (−0·02 to 0·03)	0·00 (−0·02 to 0·03)	0·00 (−0·03 to 0·03)	−0·01 (−0·04 to 0·02)	0·01 (−0·02 to 0·04)
Age 16 years	0·03 (−0·04 to 0·09)	0·02 (−0·03 to 0·07)	0·04 (−0·02 to 0·09)	−0·02 (−0·05 to 0·01)	0·01 (−0·02 to 0·03)	0·01 (−0·02 to 0·04)	−0·05 (−0·09 to −0·01)	−0·02 (−0·06 to 0·01)	0·00 (−0·04 to 0·03)
Age 23 years	−0·06 (−0·12 to 0·00)	0·01 (−0·04 to 0·07)	0·04 (−0·02 to 0·10)	−0·10 (−0·13 to −0·07)	−0·02 (−0·04 to 0·01)	0·00 (−0·02 to 0·03)	−0·07 (−0·11 to −0·04)	−0·04 (−0·08 to −0·01)	−0·01 (−0·05 to 0·02)
Age 33 years	−0·12 (−0·18 to −0·06)	−0·02 (−0·08 to 0·03)	0·01 (−0·04 to 0·06)	−0·07 (−0·10 to −0·05)	−0·01 (−0·04 to 0·01)	0·00 (−0·03 to 0·02)	−0·10 (−0·13 to −0·07)	−0·06 (−0·09 to −0·03)	−0·03 (−0·06 to −0·00)
Age 42 years	−0·13 (−0·19 to −0·07)	−0·04 (−0·09 to 0·01)	−0·01 (−0·07 to 0·04)	−0·07 (−0·10 to −0·04)	−0·01 (−0·03 to 0·02)	0·01 (−0·02 to 0·04)	−0·10 (−0·13 to −0·07)	−0·05 (−0·08 to −0·02)	−0·02 (−0·05 to 0·02)
Age 50 years	−0·10 (−0·15 to −0·04)	−0·02 (−0·07 to 0·03)	0·00 (−0·05 to 0·05)	−0·07 (−0·10 to −0·05)	−0·01 (−0·03 to 0·02)	0·01 (−0·01 to 0·04)	−0·11 (−0·14 to −0·08)	−0·05 (−0·08 to −0·02)	−0·02 (−0·05 to 0·01)
**BMI change between ages**									
Age 10–16 years	−0·04 (−0·15 to 0·07)	−0·04 (−0·13 to 0·06)	−0·02 (−0·11 to 0·08)	−0·08 (−0·12 to −0·03)	0·01 (−0·04 to 0·05)	0·02 (−0·03 to 0·06)	−0·08 (−0·13 to −0·02)	−0·03 (−0·07 to 0·02)	−0·01 (−0·06 to 0·04)
Age 16–23 years	−0·14 (−0·23 to −0·05)	0·00 (−0·08 to 0·08)	0·03 (−0·05 to 0·11)	−0·14 (−0·18 to −0·11)	−0·03 (−0·07 to 0·00)	−0·01 (−0·04 to 0·03)	−0·07 (−0·11 to −0·02)	−0·05 (−0·09 to 0·00)	−0·01 (−0·06 to 0·03)
Age 23–33 years	−0·14 (−0·22 to −0·06)	−0·05 (−0·12 to 0·02)	−0·02 (−0·09 to 0·05)	−0·01 (−0·05 to 0·03)	−0·01 (−0·05 to 0·03)	−0·01 (−0·05 to 0·03)	−0·11 (−0·17 to −0·05)	−0·07 (−0·13 to −0·01)	−0·05 (−0·10 to 0·01)
Age 33–42 years	−0·09 (−0·21 to 0·02)	−0·07 (−0·17 to 0·04)	−0·06 (−0·17 to 0·04)	−0·03 (−0·07 to 0·02)	0·01 (−0·03 to 0·05)	0·02 (−0·02 to 0·06)	−0·05 (−0·11 to 0·02)	0·00 (−0·06 to 0·06)	0·03 (−0·04 to 0·09)
Age 42–50 years	0·03 (−0·08 to 0·14)	0·02 (−0·08 to 0·12)	0·03 (−0·07 to 0·13)	−0·05 (−0·10 to −0·01)	0·00 (−0·04 to 0·04)	0·01 (−0·03 to 0·06)	−0·11 (−0·17 to −0·04)	−0·03 (−0·09 to 0·03)	−0·02 (−0·08 to 0·05)
**Duration of excess bodyweight**									
Per 10 years	−0·10 (−0·18 to −0·03)	−0·01 (−0·07 to 0·05)	0·02 (−0·04 to 0·09)	−0·09 (−0·13 to −0·07)	−0·02 (−0·04 to 0·01)	0·00 (−0·03 to 0·03)	−0·11 (−0·14 to −0·07)	−0·06 (−0·09 to −0·03)	−0·02 (−0·06 to 0·01)
**Cumulative exposure to excess bodyweight**									
Per 1 SD	−0·09 (−0·15 to −0·03)	−0·01 (−0·06 to 0·04)	0·01 (−0·05 to 0·06)	−0·07 (−0·10 to −0·05)	−0·01 (−0·04 to 0·01)	0·00 (−0·03 to 0·02)	−0·09 (−0·12 to −0·06)	−0·05 (−0·08 to −0·03)	−0·02 (−0·05 to 0·01)

Data are displayed as effect estimates and 95% CIs obtained from multivariable linear regression models with the following adjustments: model 1, adjustments for age and sex; model 2, model 1 plus additional adjustment for childhood cognitive function; model 3, model 2 plus additional adjustments for parents' BMI, child's birthweight, childhood household overcrowding, childhood socioeconomic status, and highest educational attainment achieved. All BMI exposures transformed to z-scores before model inclusion. Childhood cognitive ability was represented by a latent factor generated from harmonised measures of verbal and non-verbal ability, mathematical ability, and reading ability at age 10–11 years; midlife cognitive ability was represented by a latent factor generated from harmonised measures of immediate and delayed recall, animal naming, and letter-search speed at ages 47–53 years. NSHD=1946 Medical Research Council National Survey of Health and Development. NCDS=1958 National Child Development Study. BCS70=1970 British Cohort Study.

Midlife BMI was closely related to total duration of excess bodyweight exposure over the preceding decades (pooled analysis r=0·73; p<0·001), confirming the presence of chronic previous exposure to BMI in those reporting being overweight or obese in the midlife period. Across all three cohorts, both a longer duration and higher cumulative exposure to excess bodyweight were associated with lower midlife cognitive function in minimally adjusted models (table 2). However, similarly to previous analyses using individual BMI timepoints as exposures, these cumulative exposures attenuated towards the null once accounting for childhood cognitive function (entirely to null in the NSHD and NCDS cohorts), and fully to null following additional adjustments for other potentially confounding factors in early life. This finding was again broadly repeated when using pooled data or individual cognitive domains as outcomes (appendix pp 8, 11–13).

**Figure 2:**
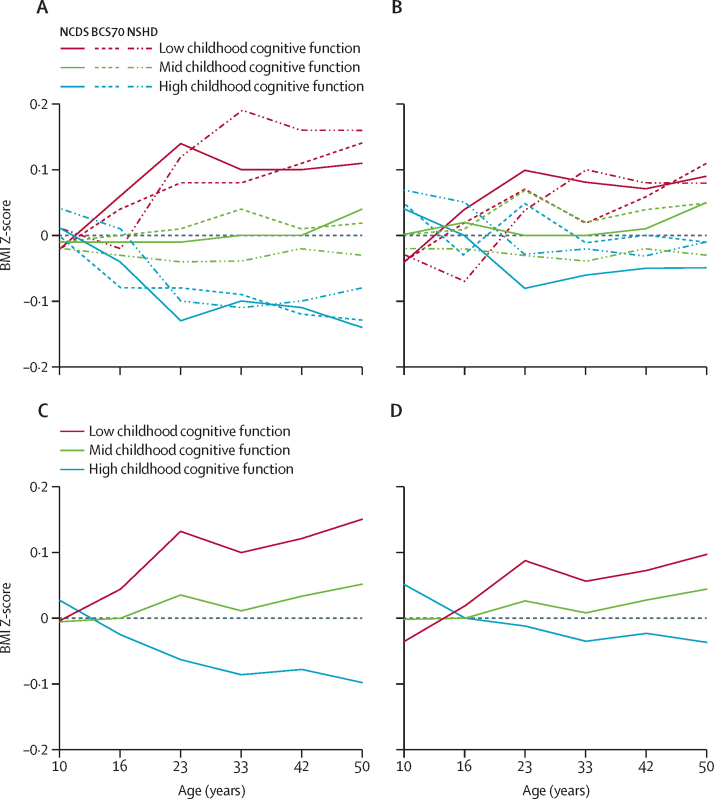
Early-life BMI trajectories categorised by tertiles of childhood cognitive function Simple line graphs connecting mean predicted values of BMI z-score at various ages across early life based on tertile of childhood cognitive function at age 10 years. Highest tertile of childhood cognitive function is indicated by blue lines, intermediate tertile is indicated by green lines, and lowest tertile is indicated by red lines. (A) NSHD, NCDS, and BCS70 adjusted for sex only. (B) NSHD, NCDS, and BCS70 adjusted for parents’ BMI, child's birthweight, childhood socioeconomic status, and childhood household overcrowding. (C) Pooled data from all three cohorts adjusted for sex. (D) Pooled data from all three cohorts adjusted for sex, parents’ BMI, child's birthweight, childhood socioeconomic status, and childhood household overcrowding. NSHD=1946 Medical Research Council National Survey of Health and Development. NCDS=1958 National Child Development Study. BCS70=1970 British Cohort Study.

We next tested the BMI and cognitive function association in the opposite direction, meaning using early-life cognitive function as an exposure and measures of later-life BMI as outcomes. Across all cohorts, being in the lowest tertile for childhood cognitive function was associated with gains in BMI across adolescence and young adulthood, followed by consistently higher BMI from young adulthood onwards. By contrast, people with the highest childhood cognitive function showed the opposite response, whereas those with intermediate cognitive function remained broadly in the centre of the distribution across the first five decades of life (figure 2). Although these effects remained following adjustment for early childhood factors such as parents’ BMI, child's birthweight, childhood household overcrowding, and childhood socioeconomic status, they were considerably attenuated, suggesting that other factors upstream of childhood cognition probably explain much of the observed associations.

**Figure 3:**
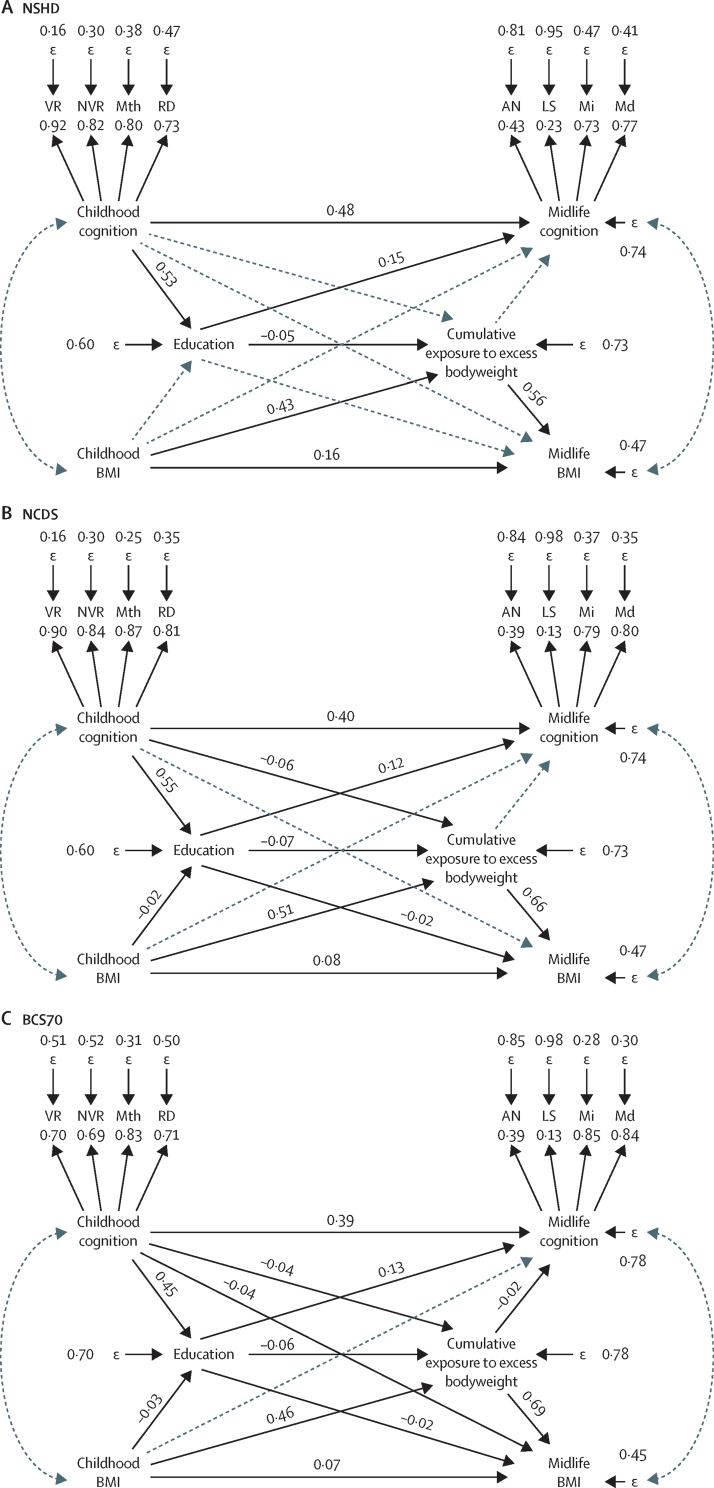
Cross-lagged structural equation models to assess potential bidirectional relationships between excess bodyweight and cognitive function during early life All variables are adjusted for sex, childhood household overcrowding, and childhood socioeconomic status. Data are presented as standardised estimates. Maximum likelihood with missing values were used to account for missing data. RMSEA was 0·06 for NSHD, 0·06 for NCDS, and 0·05 for BCS70. CFI was 0·95 for NSHD, 0·95 for NCDS, and 0·96 for BCS70. TLI was 0·91 for NSHD, 0·92 for NCDS, and 0·93 for BCS70. The dashed grey line indicates non-significant pathways; the black line indicates p<0·05. Childhood cognition was expressed as a latent variable derived from harmonised cognitive measures consisting of VR, NVR, Mth, and RD at ages 10–11 years. Midlife cognition was expressed as latent variable derived from harmonised measures of AN, LS, Mi, and Md at ages 47–53 years. NSHD=1946 Medical Research Council National Survey of Health and Development. AN=animal naming. BCS70=1970 British Cohort Study. CFI=comparative fit index. LS=letter search speed. Md=delayed recall. Mi=immediate recall. Mth=mathematical ability. NCDS=1958 National Child Development Study. NVR=non-verbal reasoning. RD=reading ability. RMSEA=root mean square error of approximation. TLI=Tucker-Lewis Index. VR=verbal reasoning.

We then tested potential bidirectional relationships between excess bodyweight and cognitive function across early life using a cross-lagged SEM. Model fit for all SEMs were good (RMSEA of 0·06 for NSHD, 0·06 for NCDS, and 0·05 for BCS70; CFI of 0·95 for NSHD, 0·95 for NCDS, and 0·96 for BCS70; and TLI of 0·91 for NSHD, 0·92 for NCDS, and 0·93 for BCS70), and models demonstrated broadly consistent associations, variance explained, and effect sizes across all three cohorts (figure 3; appendix p 14). In brief, scarce evidence was found in any cohort to support any associations between early-life excess bodyweight and midlife cognitive function, with the exception of some limited evidence in BCS70 of a significant but virtually negligible indirect pathway acting through education as a mediator. By contrast, all cohorts showed evidence of several paths connecting childhood cognitive function to later-life measures of excess bodyweight, with effect sizes similar to those observed in regression models investigating this association in the opposite direction before adjustment for early-life factors (difference in midlife BMI per 1 SD increase in childhood cognitive function approximately –0·10 for all cohorts). With the exception of a modest direct effect in the BCS70 cohort, these associations were exclusively mediated through indirect pathways involving educational achievement and cumulative exposure to excess bodyweight since childhood (figure 3; appendix p 15).

## Discussion

Our study suggests that the association between cumulative exposure to excess bodyweight in early life and lower cognitive function in midlife is probably confounded by a persistently lower cognitive function from childhood. Several findings support this conclusion. First, although all three cohorts showed evidence of lower midlife cognitive function in individuals who had experienced early and sustained weight gain, these associations were all attenuated to the null once accounting for potential childhood confounding factors, particularly childhood cognitive function. Second, when investigating this relationship in the opposite direction (ie, childhood cognition as a predictor of future excess bodyweight), we observed a similar phenomenon in reverse, in which individuals with low cognitive function in childhood were subsequently more likely to gain weight across adolescence and then maintain this excess weight across the next two or three decades of life. Further adjustments for upstream childhood disadvantage (ie, low socioeconomic position and household overcrowding) substantially attenuated these associations, however, suggesting that much of this association might be explained by other early-life factors such as family background. Third, when using cross-lagged structural equation models to test potential bidirectional relationships across four decades of life, we found little evidence to suggest any paths connecting early-life excess bodyweight to midlife cognitive function, but several direct and indirect paths (eg, via education and long-term excess bodyweight exposure) connecting childhood cognitive function to adverse cognitive and weight outcomes in later life.

The relationship between excess bodyweight, cognitive function, and dementia is complex and still not fully understood. The majority of evidence supporting the role for excess bodyweight as a major risk factor for later-life cognitive impairment comes from longitudinal observational studies, in which excess adiposity, particularly in the midlife period, has repeatedly been shown to predict dementia in later years.^7^ Large-scale observational^19^, ^20^ and mendelian randomisation^21^, ^22^, ^23^ studies have questioned the causality of this relationship, finding scarce evidence of any association (or at times even paradoxical associations) between these factors. Although the reasons for these discrepancies might arise from methodological issues with analysis such as length of follow-up or collider bias, they might also at least partly be explained by the complex causal relationships between BMI and cognition across the lifespan. In old age, it is now well established that reverse causation probably underlies the seemingly paradoxical associations between higher BMI and preserved cognitive function, insofar as progressive cognitive decline during the transition towards overt dementia probably precedes weight loss rather than the other way around.^24^ By contrast, findings from this study support and strengthen previous longitudinal studies from earlier in the lifespan which suggest that, at younger ages, the opposite of this may be true (ie, lower cognitive function during the transition to midlife probably precedes weight gain).^12^, ^25^, ^26^ We found that a persistently lower cognitive function from childhood was closely associated with the early development and then maintenance of excess bodyweight into middle age, probably explaining commonly reported associations between increased BMI and reduced cognitive function that are observed in midlife cross-sectional studies or longitudinal studies that do not have information on early-life factors.^27^, ^28^, ^29^, ^30^ These findings are likely to have implications for the interpretation of studies looking at relationships between midlife BMI and future risk of dementia, in which the assumption often made is that obesity in this supposed crucial midlife window increases dementia risk through accelerations in cognitive decline after this point. Although it is true that evidence does exist to support this phenomenon,^8^, ^10^ these rates of decline are often of a considerably lower magnitude than pre-existing differences already apparent between individuals with a healthy weight and those with excess bodyweight in the midlife baseline period. Taken together, these findings suggest that the relationship between midlife BMI and future dementia might to a large extent arise from confounding by early-life factors, such as lower childhood cognitive function, which are linked both to an increased presence of excess bodyweight in midlife and an increased risk of dementia in later life.

By contrast to the absence of association between early-life excess bodyweight and midlife cognition, we did find several lines of evidence linking these two factors in the opposite direction, meaning childhood cognition predicting weight gain. Whether or not the relationship between early-life cognitive function and excess bodyweight is causal or instead reflects other related upstream or downstream pathways, is also incompletely understood. Looking upstream of childhood cognition, we found that adjusting models for early-life childhood disadvantage had profound attenuating effects on associations between childhood cognitive function and later-life BMI, suggesting that much of this association could arise from other early-life disadvantage. These findings agree with a cross-cohort sibling comparison study,^15^ and highlight early life as a potentially important time for policy interventions which might benefit both weight status and cognitive health. Looking downstream of the childhood period, the most pronounced association between childhood cognitive function and BMI status was observed during the transition from adolescence to young adulthood, a time when young people traditionally leave both education and the parental home and begin to take more responsibility for their own decision making. By contrast, relatively little variance in BMI beyond 33 years appeared to be explained by childhood cognition, suggesting that other factors in midlife might have a greater bearing on weight status at this age. These findings suggest that, despite its association with childhood cognition and other early-life factors, excess bodyweight status in later life is probably still modifiable through the targeting of proximal factors such as education and occupational status, agreeing with previous work from these same cohorts that demonstrates a similar phenomenon in relation to midlife cognitive function.^31^ Together, these findings suggest that initiatives to improve early-life factors such as childhood disadvantage and education level are likely to have dual benefits on both physical and cognitive health well before old age.

In this study, we chose to use a latent *g* factor representing overall cognitive function in midlife as our primary outcome, a decision which might have obscured more subtle changes in distinct cognitive domains such as executive function, which have previously been suggested to be more susceptible to the negative effects of obesity.^32^ Indeed, factor loadings from SEM analyses suggested a greater contribution of immediate and delayed memory to this *g* factor, suggesting that these domains might have driven more of the observed variance in findings. Regardless, although this overall measure of cognitive function, which is analogous to general intelligence, has been suggested to be highly stable across the life course, evidence demonstrates that it remains modifiable by a wide range of early-life factors.^31^ It was therefore chosen in this study because of its relevance to future dementia risk, in which simultaneous decrements in several cognitive domains are commonly observed.^33^ Furthermore, secondary analyses of these individual domains found little evidence of any relationship between excess bodyweight status and any cognitive domain (immediate or delayed memory recall, letter-search speed, or animal naming) following adjustments for early-life factors.

There are a number of strengths and limitations to this study. First, access to three large and long-running birth cohorts containing serial measures of BMI and repeated measures of cognitive function collected at similar stages of the lifespan has allowed us to reliably reproduce our major findings across three distinct populations. It should be noted, however, that differing relationships might exist in more recent birth cohorts because of cohort effects caused by the increasing prevalence and severity of obesity since the 1970s onwards, as perhaps evidenced here by the suggestion of more pronounced, although still modest, associations between cumulative excess bodyweight and cognitive function in the BCS70 cohort. Second, although the observational nature of this study and absence of harmonised cognitive measures at all timepoints available for BMI prevents us from establishing a definitive direction of causality connecting early-life excess bodyweight and cognitive function, the availability of cumulative exposure to BMI across many decades, adjustments for important potentially confounding early-life factors often not present in many other studies, and reproducibility of findings across several distinct cohorts provides strong evidence supporting previous claims of childhood cognition as an antecedent of future excess bodyweight status rather than the other way around. Third, all three cohorts were marked by attrition between conception and midlife follow-up, resulting in a potential oversampling of individuals who are more highly educated from higher socioeconomic backgrounds. Although comparisons of early-life factors between individuals present and missing from this analysis suggested this phenomenon in the NSHD cohort, differences were less pronounced in later NCDS and BCS70 populations. Finally, it should be noted that despite finding scarce evidence of an association between cumulative exposure to excess bodyweight in early life and cognitive function by midlife, we cannot rule out a longer-term effect on subsequent cognitive impairment or dementia arising from underlying pathophysiology sustained over this period. Future studies addressing cerebral changes in early life or tracking the rate of cognitive decline in these individuals as they transition into later life might help to address this question.

In conclusion, our study suggests that interventions to reduce cumulative exposure to excess bodyweight in the early decades of life are unlikely to affect cognitive function by midlife. However, initiatives to improve early-life factors such as childhood disadvantage and education level might have dual benefits on both physical and cognitive health before old age.

## Data sharing

All data used in this publication are available to bona fide researchers upon request. NSHD data are available via a standard application procedure to the NSHD Sharing Committee, further details of which can be found at http://www.nshd.mrc.ac.uk/data. NCDS and BCS70 data are available from the UK Data Service repository at https://ukdataservice.ac.uk. The statistical code for all analyses in this paper can be found in an open-access GitHub repository located at https://github.com/scottchiesa/early-life-bmi-cog.

## Declaration of interests

We declare no competing interests.
