# Supplementary material for: Early-life cumulative exposure to excess bodyweight and midlife cognitive function: longitudinal analysis in three British birth cohorts
Source: Lancet Healthy Longev. 2024 Mar;5(3):e204–13. doi: 10.1016/S2666-7568(24)00005-9 (PMC11752840; doi:10.1016/S2666-7568(24)00005-9)
Supplement: Supplementary appendix [file mmc1.pdf]

THE LANCET
Healthy Longevity

Supplementary appendix

This appendix formed part of the original submission and has been peer reviewed. We post it as supplied by the authors.

Supplement to: Chiesa ST, Norris T, Garfield V, Richards M, Hughes AD. Early-life cumulative exposure to excess bodyweight and midlife cognitive function: longitudinal analysis in three British birth cohorts. *Lancet Healthy Longev* 2024; **5:** e204–13.

TABLE OF CONTENTS

SUPPLEMENTAL METHODS 2

Cohort Descriptions 2

Childhood Cognitive Function 2

BMI Trajectories 3

Mid-Life Cognitive Function 4

Early Life Covariates 4

SUPPLEMENTAL REFERENCES 5

SUPPLEMENTAL TABLES 6

Supplemental Table 1: Data Missingness Prior to Multiple Imputation 6

Supplemental Table 2: Participant Characteristics for Individuals With (Included in Study) and Without (Excluded from Study) Cognitive Function Measures at Age 50 7

Supplemental Table 3: Associations Between Early-Life Measures of BMI and Midlife Cognitive Outcomes in Pooled Data 8

Supplemental Table 4: Associations Between Early-Life Measures of BMI and Midlife Animal Naming and Letter Search Speed Tests 9

Supplemental Table 5: Associations Between Early-Life Measures of BMI and Midlife Immediate and Delayed Recall 10

Supplemental Table 6: Associations Between Cumulative Exposure to Overweight / Obesity Across Early-Life and Midlife Cognitive Outcomes in Pooled Data 11

Supplemental Table 7: Associations Between Cumulative Exposure to Overweight / Obesity Across Early-Life and Midlife Animal Naming and Letter Search Speed Tests 12

Supplemental Table 8: Associations Between Cumulative Exposure to Overweight / Obesity Across Early-Life and Midlife Immediate and Delayed Recall 13

Supplemental Table 9: Variance of Each Midlife Outcome Explained by Structural Equation Models 14

Supplemental Table 10: Total, Direct, and Indirect Bidirectional Pathways Linking Childhood BMI to Midlife Cognitive Function and Childhood Cognitive Function to Midlife BMI in Structural Equation Models 15

SUPPLEMENTAL FIGURES 16

Supplemental Figure 1: Schematic of Study Design 16

SUPPLEMENTAL METHODS

Cohort Descriptions

The NSHD is a socially stratified birth cohort of 2,547 women and 2,815 men and is a sample of all births in England, Scotland, and Wales that occurred in one week in March 1946. It consists of all single births to married women with a husband in non-manual and agricultural employment, and 1 in 4 of all comparable births to women with a husband in manual employment[1]. The NCDS follows the lives of 17415 people that were born in England, Scotland or Wales in a single week in March 1958, starting in 1958 as the Perinatal Mortality Survey and capturing 98% of total births in Great Britain in the target week. The cohort consists of a total of 11 sweeps between birth and 55, with a 12th sweep at age 62 currently in progress and scheduled for completion in 2024[2]. The BCS70 follows the lives of 17,198 people born in England, Scotland and Wales in a single week in March 1970. The cohort consists of 10 sweeps between birth and age 47, with an 11th sweep at age 51 currently in progress and anticipated for completion in 2023[3].

Childhood Cognitive Function

Brief descriptions of each test can be found below. More detailed information on each test and how these were harmonised can be found in main manuscript reference[16].

Verbal Reasoning in both NSHD and NCDS was measured using the National Foundation for Education Research (NFER) Verbal Ability Test, in which children were asked to select 40 words to match groups of four other words linked either logically, semantically, or phonologically. In BCS70, a similar construct was measured using the British Ability Scale (BAS) Word Similarities Test consisting on 21 items made up of three linked words, with children asked to name the 4th example and describe the commonalty underlying items.

Non-Verbal Reasoning in both NSHD and NCDS was measured using the NFER Non-Verbal Ability Test, in which children were asked to select 40 shapes to match groups of four other linked shapes. In BCS70, the

BAS Matrices Test was used instead, in which a blank square in the lower right corner of a grid of four or nine cells was to be filled in with an appropriate shape.

Mathematic Ability in NSHD was measured using the NFER Arithmetic Test, comprising 50 questions (20 sums, 30 problems) assessing ability to add, subtract, multiply, and divide. In NCDS a mathematics test comprising 40 questions testing number skills, fractions, measures and geometry was used, while in BCS70 the Friendly Maths Test consisting of 72 questions of increasing difficulty was used.

Reading Ability in NSHD was assessed using a vocabulary test in which the child was asked for the meaning of each of 50 words read as previous reading task. In NCDS, the NFER Reading Comprehension Test was used, where the child was required to read 35 sentences and choose from a selection of 5 words the most appropriate word to complete the sentence. In BCS70, the Pictorial Language Comprehension Test was used, where children were required to indicate correct word describing each of 100 sets of 4 pictures.

BMI Trajectories

BMI trajectories were modelled using restricted cubic splines with mixed effects, resulting in the estimation of annual BMI values between the ages of 10-40 years for each individual. Using these fitted BMI values, z-scores were then created relative to the International Obesity Task Force (IOTF) reference[4]. O/O was defined as a z-score of >1.35 in males and >1.24 in females, which corresponds to a BMI value of 25 kg/m^2 at 18 years. Using the sex-specific O/O cut-off, total duration of O/O was calculated as the length of time in years that an individual's BMI z-score exceeded this O/O threshold; with this value included in later models both continuously and following stratification into six categories of roughly 5-year durations. A composite trapezoid rule was then used to calculate the area under the curve for time spent above the O/O threshold, with this measure taken to represent a metric of cumulative exposure to O/O incorporating both severity of BMI exposure and time spent in an O/O state.

Mid-Life Cognitive Function

Immediate Verbal Recall in NSHD was assessed by asking participants to write down as many words as possible after attempting to memorise a list of 15 viewed words. Any scores > 10 were subsequently recoded to 10 to match other cohorts. In NCDS and BCS70, participants had 2 minutes to recall as many words as possible from an audio recording of 10 words.

Delayed Verbal Recall in all cohorts was assessed by asking participants to once again recall as many words as possible later in the cognitive battery.

Animal Naming Verbal Fluency in all cohorts was tested by asking participants to name as many animals as possible in one minute.

Letter Search Speed Participants were asked to scan blocks of letters from left to right crossing out all Ps and Ws as quickly and accurately as possible. Search speed was calculated as total words scanned within the time limit allowed.

Early Life Covariates

Parents BMI in all cohorts was calculated as weight/height^2. Height and weight of both parents in NSHD were reported when the child was 6 years old. In NCDS, the weight of both parents was measured when study participants were 11 years old and categorised into one of 27 groups of 6lb increments (covering roughly 6.5 stone to 20 stone). These groupings were converted to kilograms and then combined with measured height (collected concurrently in fathers and retrospectively from earlier measures in mothers) to calculate BMI. In BCS70, height and weight of both parents were reported by the study participant's mother when the child was age 10. Birthweight was extracted from records created within the first week of life by the midwife or senior midwife involved in the child's delivery. Household overcrowding was reported as average number of people per room during childhood. Childhood socioeconomic status in all cohorts was recorded at ~10 years old based on father's occupational status according to the Registrar

General’s Social Class System. Finally, highest educational attainment was assessed at age 26 in NSHD, age 33 in NCDS, and age 30 in BCS70. In all cohorts, these data were again harmonised to represent five categories of educational achievements based on UK National Vocational Qualification (NVQ) categories (0 = no education to 5 = postgraduate or above).

SUPPLEMENTAL REFERENCES

1 Kuh D, Pierce M, Adams J, *et al.* Cohort profile: updating the cohort profile for the MRC National Survey of Health and Development: a new clinic-based data collection for ageing research. *Int J Epidemiol* 2011; **40**: e1-9.

2 Power C, Elliott J. Cohort profile: 1958 British birth cohort (National Child Development Study). *International Journal of Epidemiology* 2006; **35**: 34–41.

3 Sullivan A, Brown M, Hamer M, Ploubidis GB. Cohort Profile Update: The 1970 British Cohort Study (BCS70). *Int J Epidemiol* 2022; : dyac148.

4 Cole TJ, Bellizzi MC, Flegal KM, Dietz WH. Establishing a standard definition for child overweight and obesity worldwide: international survey. *BMJ* 2000; **320**: 1240.

SUPPLEMENTAL TABLES

Supplemental Table 1: Data Missingness Prior to Multiple Imputation

	NSHD			NCDS			BCS70		
**Variable**	**Complete**	**Imputed**	**% Missing**	**Complete**	**Imputed**	**% Missing**	**Complete**	**Imputed**	**% Missing**
Age 10 BMI	1788	343	16	7399	1986	21	6476	1750	21
Age 16 BMI	1624	507	24	6705	2680	29	3521	4705	57
Age 23 BMI	1676	455	21	7860	1525	16	4666	3560	43
Age 33 BMI	1917	214	10	7864	1521	16	6520	1706	20
Age 42 BMI	1987	144	7	8561	824	9	6199	2027	25
Age 50 BMI	2110	21	1	7533	1852	20	7177	1049	13
Mother's BMI	1855	276	13	7834	1551	17	6979	1247	15
Father's BMI	1461	670	31	7617	1768	19	6645	1581	19
Birthweight	2122	9	0	8606	779	8	5325	2901	35
Childhood Cognition	1832	299	14	8142	1243	13	5965	2261	27
Childhood Overcrowding	2011	120	6	7914	1471	16	6662	1564	19
Childhood Socioeconomic Status	1822	309	15	6360	3025	32	6507	1719	21
Highest Education Level Attained	1580	551	26	7974	1411	15	7119	1107	13

Supplemental Table 2: Participant Characteristics for Individuals With (Included in Study) and Without (Excluded from Study) Cognitive Function Measures at Age 50

	NSHD		NCDS		BCS70	
	Mean ± SD / Median (IQR)		Mean ± SD / Median (IQR)		Mean ± SD / Median (IQR)	
**Variable**	***Included (n=2131)***	***Excluded (n=3229)***	***Included (n=9385)***	***Excluded (n=8030)***	***Included (n=8226)***	***Excluded (n=8972)***
Sex (% female)	49	47	51	46	52	44
Age 10 BMI (kg/m^2)	17.0 (15.8 – 18.4)	16.9 (15.8 – 18.4)	16.9 (15.8 – 18.5)	16.9 (15.8 – 18.5)	16.5 (15.5 – 17.9)	16.5 (15.5 – 17.9)
Age 16 BMI (kg/m^2)	19.7 (18.2 – 21.5)	19.6 (18.3 – 21.5)	20.2 (18.8 – 22.0)	20.2 (18.7 – 21.9)	20.7 (19.2 – 22.8)	20.8 (19.1 – 22.7)
Age 23 BMI (kg/m^2)	22.0 (20.4 – 23.8)	22.0 (20.4 – 23.6)	22.1 (20.5 – 24.0)	22.2 (20.5 – 24.2)	23.0 (21.1 – 25.4)	22.8 (21.0 – 25.4)
Age 33 BMI (kg/m^2)	23.8 (21.8 – 26.2)	23.6 (21.5 – 25.9)	24.3 (22.2 – 27.1)	24.5 (22.2 – 27.4)	25.0 (22.6 – 28.0)	25.2 (22.6 – 28.6)
Age 42 BMI (kg/m^2)	24.8 (22.7 – 27.6)	24.7 (22.5 – 27.4)	25.2 (22.8 – 28.1)	25.4 (22.8 – 28.2)	25.9 (23.3 – 29.3)	26.0 (23.3 – 29.8)
Age 50 BMI (kg/m^2)	26.7 (24.2 – 29.9)	26.6 (24.1 – 29.8)	26.7 (24.0 – 30.1)	26.9 (23.5 – 30.4)	27.8 (24.6 – 31.5)	27.7 (24.4 – 32.6)
Mother's BMI (kg/m^2)	23.0 (20.7 – 25.8)	22.8 (20.8 – 25.2)	23.0 (21.3 – 25.6)	23.2 (21.2 – 26.0)	22.5 (20.9 – 24.8)	22.7 (21.1 – 25.3)
Father's BMI (kg/m^2)	23.7 (22.1 – 25.8)	23.6 (22.0 – 25.7)	24.5 (22.6 – 26.4)	24.5 (22.6 – 26.6)	24.0 (22.4 – 25.9)	24.3 (22.5 – 26.3)
Birthweight (kg)	3.4 ± 0.5	3.3 ± 0.6	3.3 ± 0.5	3.3 ± 0.6	3.3 ± 0.5	3.3 ± 0.6
Childhood Cognition						
- Verbal reasoning	30 ± 11	29 ± 12	29 ± 11	25 ± 12	30 ± 6	29 ± 6
- Non-verbal reasoning	28 ± 9	27 ± 10	28 ± 9	24 ± 10	29 ± 9	26 ± 9
- Mathematic ability	27 ± 11	26 ± 12	23 ± 13	18± 13	32 ± 8	29 ± 9
- Reading ability	30 ± 7	30 ± 8	24 ± 9	21 ± 9	31 ± 5	30 ± 5
Household Overcrowding (persons per room - %)						
- Up to 1	57	56	60	54	85	79
- Over 1 to 1.5	27	24	27	29	12	17
- Over 1.5 to 2	10	11	10	13	2	3
- Over 2	6	9	3	4	1	1
Childhood Socioeconomic Status (%)						
- V unskilled	8	9	5	8	4	6
- IV partly-skilled	19	18	14	17	12	15
- III skilled	49	48	53	55	53	56
- II managerial and technical	19	16	22	16	22	17
- I professional	6	6	6	4	8	6
Highest Education Level Attained (%)						
- Below ordinary secondary	44	42	22	33	31	43
- Ordinary secondary	20	45	49	46	38	37
- Advanced level	27	12	15	12	7	6
- Higher	9	1	14	9	24	14

Supplemental Table 3: Associations Between Early-Life Measures of BMI and Midlife Cognitive Outcomes in Pooled Data

	Pooled (n=19,609)					
	**Model 1**		**Model 2**		**Model 3**	
	**Beta (95%CI)**	**p**	**Beta (95%CI)**	**p**	**Beta (95%CI)**	**p**
BMI at Distinct Ages						
Age 10	0.01 (-0.01, 0.03)	0.207	0.00 (-0.01, 0.02)	0.632	0.01 (-0.01, 0.03)	0.174
Age 16	**-0.02 (-0.05, 0.00)**	**0.024**	0.00 (-0.02, 0.02)	0.696	0.01 (-0.01, 0.03)	0.535
Age 23	**-0.08 (-0.10, -0.06)**	**<0.001**	**-0.02 (-0.04, -0.01)**	**0.013**	0.00 (-0.02, 0.02)	0.849
Age 33	**-0.09 (-0.11, -0.07)**	**<0.001**	**-0.04 (-0.05, -0.02)**	**<0.001**	-0.01 (-0.03, 0.00)	0.129
Age 42	**-0.09 (-0.11, -0.07)**	**<0.001**	**-0.03 (-0.05, -0.01)**	**0.002**	-0.01 (-0.02, 0.01)	0.540
Age 50	**-0.09 (-0.11, -0.07)**	**<0.001**	**-0.03 (-0.04, -0.01)**	**0.002**	0.00 (-0.02, 0.01)	0.643
BMI Change Between Ages						
Age 10-16	**-0.06 (-0.09, -0.03)**	**<0.001**	-0.04 (-0.04, 0.02)	0.362	0.00 (-0.03, 0.02)	0.764
Age 16-23	**-0.12 (-0.15, -0.09)**	**<0.001**	**-0.04 (-0.06, -0.01)**	**0.005**	-0.01 (-0.04, 0.02)	0.470
Age 23-33	**-0.06 (-0.09, 0.03)**	**<0.001**	**-0.04 (-0.07, -0.01)**	**0.010**	-0.03 (-0.06, 0.00)	0.067
Age 33-42	**-0.04 (-0.08, -0.01)**	**0.021**	0.00 (-0.04, 0.03)	0.800	0.01 (-0.02, 0.05)	0.386
Age 42-50	**-0.08 (-0.11, -0.03)**	**<0.001**	-0.01 (-0.05, 0.02)	0.522	0.00 (-0.03, 0.03)	0.956

Results displayed as effect estimates and 95% confidence intervals obtained from mixed linear models with cohort included as random effect and following adjustments: Model 1 = adjustments for age and sex; Model 2 = Model 1 + additional adjustment for childhood cognitive function; Model 3 = Model 2 + additional adjustments for parents BMI, birthweight, childhood household overcrowding, childhood SES, and highest educational attainment achieved.

Supplemental Table 4: Associations Between Early-Life Measures of BMI and Midlife Animal Naming and Letter Search Speed Tests

	Animal Naming						*Letter Search Speed*					
	**NSHD**		**NCDS**		**BCS70**		**NSHD**		**NCDS**		**BCS70**	
	**Beta (95%CI)**	**p**	**Beta (95%CI)**	**p**	**Beta (95%CI)**	**p**	**Beta (95%CI)**	**p**	**Beta (95%CI)**	**p**	**Beta (95%CI)**	**p**
BMI at Distinct Ages												
Age 10	-0.01 (-0.07, 0.06)	0.832	0.00 (-0.03, 0.03)	0.987	**0.04 (0.00, 0.07)**	**0.046**	**0.08 (0.00, 0.15)**	**0.036**	-0.02 (-0.06, 0.02)	0.264	0.01 (-0.02, 0.05)	0.523
Age 16	-0.02 (-0.08, 0.05)	0.662	0.01 (-0.02, 0.05)	0.398	-0.02 (-0.06, 0.03)	0.450	0.07 (-0.01, 0.14)	0.070	0.02 (-0.01, 0.06)	0.186	-0.03 (-0.08, 0.02)	0.199
Age 23	0.01 (-0.05, 0.08)	0.695	0.01 (-0.03, 0.04)	0.639	0.00 (-0.04, 0.05)	0.867	0.03 (-0.04, 0.10)	0.390	0.03 (-0.01, 0.07)	0.098	0.00 (-0.04, 0.05)	0.887
Age 33	-0.02 (-0.08, 0.05)	0.631	0.01 (-0.02, 0.04)	0.474	-0.02 (-0.05, 0.02)	0.397	0.03 (-0.04, 0.10)	0.409	0.02 (-0.02, 0.05)	0.335	0.00 (-0.04, 0.04)	0.912
Age 42	0.03 (-0.03, 0.10)	0.291	0.03 (-0.01, 0.06)	0.111	-0.02 (-0.06, 0.02)	0.242	-0.04 (-0.10, 0.03)	0.280	0.02 (-0.01, 0.05)	0.241	0.02 (-0.02, 0.06)	0.287
Age 46	0.02 (-0.05, 0.07)	0.632	0.00 (-0.03, 0.03)	0.943	0.00 (-0.04, 0.03)	0.943	-0.03 (-0.10, 0.03)	0.280	0.00 (-0.03, 0.04)	0.788	0.00 (-0.04, 0.03)	0.809
BMI Change Between Ages												
Age 10-16	-0.04 (-0.11, 0.04)	0.330	0.02 (-0.01, 0.06)	0.161	0.01 (-0.02, 0.04)	0.553	0.00 (-0.08, 0.07)	0.902	0.03 (0.00, 0.07)	0.051	-0.01 (-0.05, 0.02)	0.546
Age 16-23	0.03 (-0.03, 0.09)	0.331	0.00 (-0.02, 0.03)	0.795	-0.01 (-0.04, 0.02)	0.610	0.01 (-0.05, 0.07)	0.798	-0.01 (-0.04, 0.02)	0.644	-0.01 (-0.04, 0.03)	0.694
Age 23-33	-0.05 (-0.10, 0.01)	0.112	0.00 (-0.03, 0.03)	0.844	-0.01 (-0.05, 0.03)	0.721	-0.03 (-0.09, 0.03)	0.322	0.00 (-0.03, 0.03)	0.932	-0.02 (-0.06, 0.02)	0.323
Age 33-42	0.05 (-0.03, 0.13)	0.195	0.02 (-0.01, 0.05)	0.193	0.02 (-0.03, 0.06)	0.398	**-0.10 (-0.18, -0.01)**	**0.026**	0.00 (-0.03, 0.03)	0.927	0.04 (-0.01, 0.08)	0.103
Age 42-46	0.00 (-0.08, 0.08)	0.990	0.00 (-0.03, 0.03)	0.905	-0.01 (-0.05, 0.04)	0.728	0.01 (-0.07, 0.10)	0.715	0.01 (-0.03, 0.04)	0.772	-0.01 (-0.05, 0.04)	0.796

Results displayed as effect estimates and 95% confidence intervals obtained from multivariable linear regression models adjusted for age, sex, childhood cognitive function, parents BMI, birthweight, childhood household overcrowding, childhood SES, and highest educational attainment achieved.

Supplemental Table 5: Associations Between Early-Life Measures of BMI and Midlife Immediate and Delayed Recall

	Immediate Recall						*Delayed Recall*					
	**NSHD**		**NCDS**		**BCS70**		**NSHD**		**NCDS**		**BCS70**	
	**Beta (95%CI)**	**p**	**Beta (95%CI)**	**p**	**Beta (95%CI)**	**p**	**Beta (95%CI)**	**p**	**Beta (95%CI)**	**p**	**Beta (95%CI)**	**p**
BMI at Distinct Ages												
Age 10	0.00 (-0.06, 0.07)	0.968	-0.01 (-0.04, 0.03)	0.759	0.01 (-0.03, 0.04)	0.763	0.02 (-0.04, 0.08)	0.462	0.00 (-0.03, 0.03)	0.936	0.02 (-0.01, 0.06)	0.198
Age 16	0.03 (-0.04, 0.09)	0.412	0.00 (-0.03, 0.03)	0.808	-0.04 (-0.08, 0.01)	0.127	**0.07 (0.01, 0.13)**	**0.030**	0.00 (-0.03, 0.03)	0.990	-0.01 (-0.06, 0.04)	0.638
Age 23	-0.03 (-0.09, 0.04)	0.404	-0.03 (-0.06, 0.01)	0.147	0.00 (-0.04, 0.05)	0.934	0.01 (-0.05, 0.07)	0.742	-0.01 (-0.05, 0.02)	0.411	0.00 (-0.05, 0.04)	0.909
Age 33	0.02 (-0.04, 0.08)	0.564	**-0.04 (-0.07, -0.01)**	**0.019**	-0.01 (-0.05, 0.03)	0.609	0.06 (-0.01, -0.12)	0.073	-0.01 (-0.04, 0.02)	0.494	-0.01 (-0.05, 0.03)	0.530
Age 42	0.02 (-0.04, 0.09)	0.464	-0.02 (-0.05, 0.02)	0.327	0.00 (-0.04, 0.04)	0.942	-0.03 (-0.03, 0.09)	0.270	0.00 (-0.03, 0.03)	0.841	-0.01 (-0.05, 0.03)	0.636
Age 46	-0.01 (-0.07, 0.04)	0.665	-0.02 (-0.06, 0.01)	0.175	0.00 (-0.04, 0.04)	0.994	0.03 (-0.03, 0.08)	0.369	0.00 (-0.03, 0.03)	0.940	0.00 (-0.04, 0.03)	0.870
BMI Change Between Ages												
Age 10-16	0.00 (-0.08, 0.07)	0.932	0.00 (-0.03, 0.03)	0.891	-0.01 (-0.04, 0.03)	0.637	0.01 (-0.07, 0.08)	0.847	0.00 (-0.03, 0.04)	0.895	-0.01 (-0.05, 0.02)	0.448
Age 16-23	-0.01 (-0.07, 0.06)	0.846	-0.01 (-0.04, 0.02)	0.412	0.00 (-0.03, 0.03)	0.952	0.03 (-0.03, 0.09)	0.343	0.00 (-0.03, 0.03)	0.945	-0.01 (-0.04, 0.02)	0.506
Age 23-33	-0.02 (-0.08, 0.04)	0.478	-0.01 (-0.04, 0.02)	0.386	-0.03 (-0.06, 0.01)	0.158	0.03 (-0.03, 0.08)	0.316	0.00 (-0.03, 0.03)	0.952	-0.04 (-0.08, 0.00)	0.062
Age 33-42	-0.06 (-0.14, 0.02)	0.121	0.01 (-0.02, 0.04)	0.483	0.01 (-0.04, 0.05)	0.751	-0.04 (-0.12, 0.04)	0.343	0.01 (-0.02, 0.04)	0.454	0.01 (-0.03, 0.05)	0.699
Age 42-46	-0.02 (-0.09, 0.06)	0.626	0.01 (-0.03, 0.04)	0.707	-0.02 (-0.06, 0.03)	0.468	0.06 (-0.01, 0.13)	0.115	0.01 (-0.02, 0.05)	0.413	0.00 (-0.05, 0.04)	0.852

Results displayed as effect estimates and 95% confidence intervals obtained from multivariable linear regression models adjusted for age, sex, childhood cognitive function, parents BMI, birthweight, childhood household overcrowding, childhood SES, and highest educational attainment achieved.

Supplemental Table 6: Associations Between Cumulative Exposure to Overweight / Obesity Across Early-Life and Midlife Cognitive Outcomes in Pooled Data

	Pooled (n=19,609)					
	**Model 1**		**Model 2**		**Model 3**	
	**Beta (95%CI)**	**p**	**Beta (95%CI)**	**p**	**Beta (95%CI)**	**p**
Duration of Overweight / Obesity						
Per 10 years	**-0.10 (-0.12, -0.08)**	**<0.001**	**-0.04 (-0.06, -0.02)**	**0.001**	0.00 (-0.03, 0.01)	0.380
Cumulative Exposure to Overweight/Obesity Between Ages 10 - 40						
Per 1SD	**-0.08 (-0.10, -0.06)**	**<0.001**	**-0.03 (-0.05, -0.01)**	**<0.001**	-0.01 (-0.03, 0.01)	0.174

Results displayed as effect estimates and 95% confidence intervals obtained from mixed linear models with cohort included as random effect and following adjustments: Model 1 = adjustments for age and sex; Model 2 = Model 1 + additional adjustment for childhood cognitive function; Model 3 = Model 2 + additional adjustments for parents BMI, birthweight, childhood household overcrowding, childhood SES, and highest educational attainment achieved.

Supplemental Table 7: Associations Between Cumulative Exposure to Overweight / Obesity Across Early-Life and Midlife Animal Naming and Letter Search Speed Tests

	Animal Naming						*Letter Search Speed*					
	**NSHD**		**NCDS**		**BCS70**		**NSHD**		**NCDS**		**BCS70**	
	**Beta (95%CI)**	**p**	**Beta (95%CI)**	**p**	**Beta (95%CI)**	**p**	**Beta (95%CI)**	**p**	**Beta (95%CI)**	**p**	**Beta (95%CI)**	**p**
Duration of Overweight/Obesity												
Per 10 years	0.00 (-0.07, 0.08)	0.973	0.01 (-0.02, 0.04)	0.655	0.00 (-0.04, 0.03)	0.887	0.03 (-0.04, -0.11)	0.390	0.01 (-0.03, 0.05)	0.605	-0.01 (-0.05, 0.03)	0.480
Cumulative Exposure to Overweight/Obesity Between Ages 10 - 40												
Per 1SD	0.01 (-0.05, 0.07)	0.662	0.01 (-0.02, 0.04)	0.524	-0.01 (-0.04, 0.03)	0.579	0.00 (-0.06, 0.06)	0.941	0.00 (-0.03, 0.04)	0.897	0.00 (-0.03, 0.04)	0.913

Results displayed as effect estimates and 95% confidence intervals obtained from multivariable linear regression models adjusted for age, sex, childhood cognitive function, parents BMI, birthweight, childhood household overcrowding, childhood SES, and highest educational attainment achieved.

Supplemental Table 8: Associations Between Cumulative Exposure to Overweight / Obesity Across Early-Life and Midlife Immediate and Delayed Recall

	Immediate Recall						***Delayed Recall***					
	**NSHD**		**NCDS**		**BCS70**		**NSHD**		**NCDS**		**BCS70**	
	**Beta (95%CI)**	**p**	**Beta (95%CI)**	**p**	**Beta (95%CI)**	**p**	**Beta (95%CI)**	**p**	**Beta (95%CI)**	**p**	**Beta (95%CI)**	**p**
Duration of Overweight/Obesity												
Per 10 years	-0.02 (-0.09, 0.06)	0.678	-0.03 (-0.06, 0.00)	0.083	-0.02 (-0.05, 0.02)	0.404	0.05 (-0.02, 0.12)	0.147	-0.01 (-0.05, 0.02)	0.432	-0.02 (-0.06, 0.02)	0.350
Cumulative Exposure to Overweight/Obesity Between Ages 10 - 40												
Per 1SD	0.00 (-0.06, 0.05)	0.895	**-0.03 (-0.06, 0.00)**	**0.040**	0.00 (-0.03, 0.04)	0.946	0.03 (-0.03, 0.08)	0.320	-0.02 (-0.05, 0.01)	0.273	-0.01 (-0.05, 0.02)	0.560

Results displayed as effect estimates and 95% confidence intervals obtained from multivariable linear regression models adjusted for age, sex, childhood cognitive function, parents BMI, birthweight, childhood household overcrowding, childhood SES, and highest educational attainment achieved

Supplemental Table 9: Variance of Each Midlife Outcome Explained by Structural Equation Models

	R^2	
	**Midlife Cognition**	**Midlife BMI**
**NSHD**	0.40	0.43
**NCDS**	0.27	0.53
**BCS70**	0.23	0.55

NSHD = 1946 Medical Research Council National Survey of Health and Development; NCDS = 1958 National Child Development Study; BCS70 = 1970 British Cohort Study.

Supplemental Table 10: Total, Direct, and Indirect Bidirectional Pathways Linking Childhood BMI to Midlife Cognitive Function and Childhood Cognitive Function to Midlife BMI in Structural Equation Models

			Childhood BMI and Midlife Cognitive Function		Childhood Cognitive Function and Midlife BMI	
			**Beta (95% CI)**	**p-value**	**Beta (95% CI)**	**p-value**
**Total Effects**	**Exposure -> Outcome**	**NSHD**	0.03 (-0.02, 0.07)	0.238	**-0.07 (-0.12, -0.02)**	**0.005**
		**NCDS**	0.01 (-0.02, 0.03)	0.569	**-0.09 (-0.11, -0.06)**	**< 0.001**
		**BCS70**	-0.01 (-0.04, 0.01)	0.372	**-0.10 (-0.12, -0.07)**	**< 0.001**
**Direct Effects**	**Exposure -> Outcome**	**NSHD**	0.02 (-0.03, 0.08)	0.365	0.00 (-0.05, 0.05)	0.899
		**NCDS**	0.01 (-0.01, 0.04)	0.310	-0.02 (-0.04, 0.01)	0.178
		**BCS70**	0.00 (-0.02, 0.03)	0.790	**-0.04 (-0.06, -0.01)**	**0.004**
**Indirect Effects**	**Exposure -> Outcome**	**NSHD**	0.00 (-0.02, 0.03)	0.731	**-0.07 (-0.10, -0.03)**	**0.001**
		**NCDS**	-0.01 (-0.02, 0.01)	0.267	**-0.07 (-0.09, -0.05)**	**< 0.001**
		**BCS70**	**-0.01 (-0.03, 0.00)**	**0.016**	**-0.06 (-0.08, -0.04)**	**< 0.001**
**Specific Indirect Effects**	**Exposure -> Education -> Outcome**	**NSHD**	0.00 (0.00, 0.01)	0.293	-0.02 (-0.05, 0.01)	0.131
		**NCDS**	**0.00 (0.00, 0.00)**	**0.023**	**-0.01 (-0.03, 0.00)**	**0.036**
		**BCS70**	**0.00 (-0.01, 0.00)**	**0.018**	**-0.01 (-0.02, 0.00)**	**0.028**
	**Exposure -> Cumulative O/O -> Outcome**	**NSHD**	0.00 (-0.02, 0.02)	0.967	-0.03 (-0.06, 0.00)	0.076
		**NCDS**	0.00 (-0.02, 0.01)	0.481	**-0.04 (-0.05, -0.02)**	**< 0.001**
		**BCS70**	-0.01 (-0.02, 0.00)	0.061	**-0.03 (-0.05, -0.01)**	**0.007**
	**Exposure -> Education -> Cumulative O/O -> Outcome**	**NSHD**	0.00 (0.00, 0.00)	0.967	-0.01 (-0.03, 0.00)	0.116
		**NCDS**	0.00 (0.00, 0.00)	0.504	**-0.02 (-0.03, -0.01)**	**< 0.001**
		**BCS70**	0.00 (0.00, 0.00)	0.153	**-0.02 (-0.03, -0.01)**	**< 0.001**

Results displayed as effect estimates and (5% confidence intervals obtained from structural equation models. All estimates adjusted for sex, childhood household overcrowding, and childhood SES. Data presented as standardised estimates. Maximum likelihood with missing values used to account for missing data. Bold text represents associations with p < 0.05. NSHD = 1946 Medical Research Council National Survey of Health and Development; NCDS = 1958 National Child Development Study; BCS70 = 1970 British Cohort Study.

SUPPLEMENTAL FIGURES

Supplemental Figure 1: Schematic of Study Design

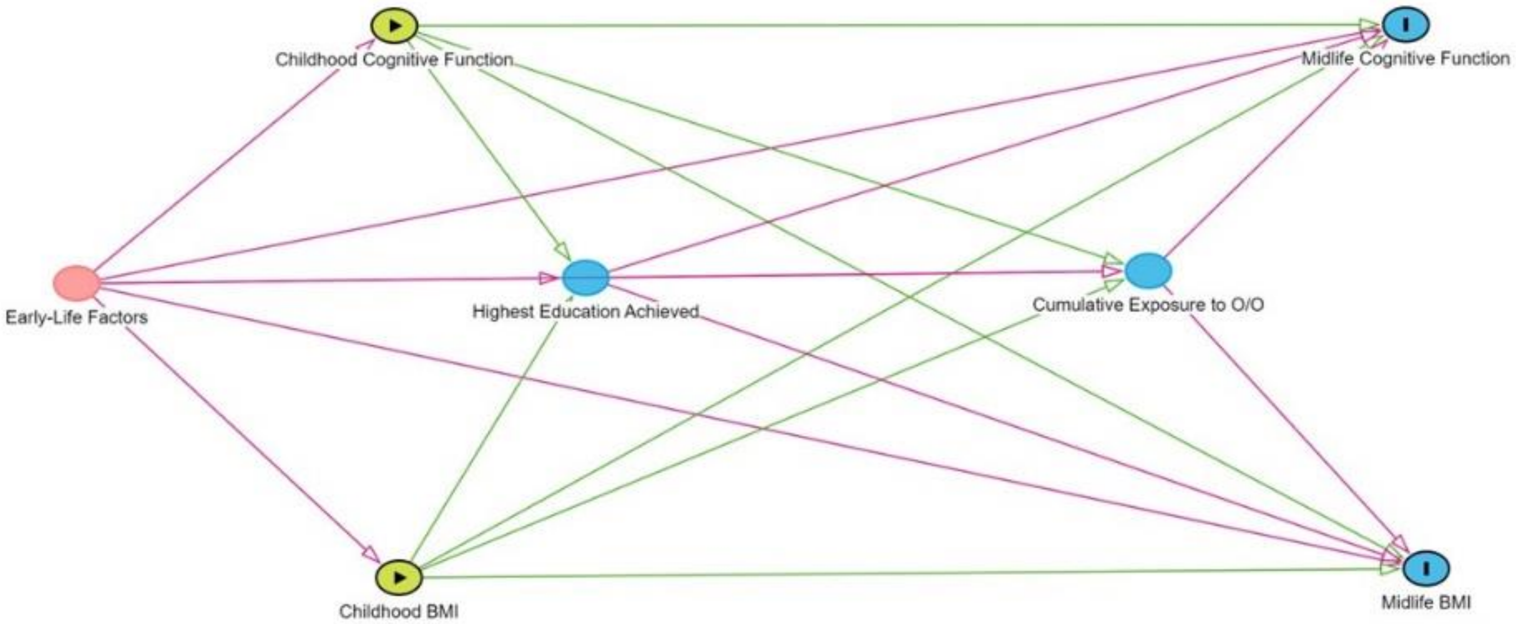

Cohorts	NSHD / NCDS / BCS70			
Stage of Lifespan	Early-Life	Age 10	Adolescence / Young Adulthood /Adulthood	Age 50
Variables	Parents BMI / Birthweight / Sex / Childhood SES / Childhood Overcrowding	Childhood Cognitive Function / Childhood BMI	Highest Education Level Achieved / BMI Age 16 / BMI Age 23 / BMI Age 33 / BMI Age 42	Midlife Cognitive Function / Midlife BMI
Analyses	Associations Between Early-Life BMI and Midlife Cognitive Function	Table 2	Multivariable linear regression models	
	Associations Between Childhood Cognitive Function and Early-Life BMI	Figure 2	Trajectories of BMI deviations over time	
	Bidirectional Relationships Between O/O and Cognitive Function Across Early-Life	Figure 3	Cross-lagged structural equation models (SEM)	
